# Numerical renormalization group study of the Loschmidt echo in Kondo systems

**DOI:** 10.1038/s41598-022-14108-x

**Published:** 2022-06-13

**Authors:** Tomasz Ślusarski, Kacper Wrześniewski, Ireneusz Weymann

**Affiliations:** Institute of Spintronics and Quantum Information, Faculty of Physics, Adam Mickiewicz University, Uniwersytetu Poznańskiego 2, Poznań, 61-614 Poland

**Keywords:** Electronic devices, Molecular electronics

## Abstract

We study the dynamical properties of the one-channel and two-channel spin-1/2 Kondo models after quenching in Hamiltonian variables. Eigen spectrum of the initial and final Hamiltonians is calculated by using the numerical renormalization group method implemented within the matrix product states formalism. We consider multiple quench protocols in the considered Kondo systems, also in the presence of external magnetic field of different intensities. The main emphasis is put on the analysis of the behavior of the Loschmidt echo *L*(*t*), which measures the ability of the system’s revival to its initial state after a quench. We show that the decay of the Loschmidt echo strongly depends on the type of quench and the ground state of the system. For the one-channel Kondo model, we show that *L*(*t*) decays as, $L(t)\sim (t\cdot T_K)^{-1.4}$, where $T_K$ is the Kondo temperature, while for the two-channel Kondo model, we demonstrate that the decay is slower and given by $L(t)\sim (t\cdot T_K)^{-0.7}$. In addition, we also determine the dynamical behavior of the impurity’s magnetization, which sheds light on identification of the relevant time scales in the system’s dynamics.

## Introduction

Dynamical properties of quantum impurity systems are of particular interest in recent years due to rapid development of spintronics and, in general, nanoscience ^1^. Tracking the behavior of impurity as a function of time is crucial in the development of real systems relying on stabilization, manipulation and control of local degrees of freedom ^2^, such as (pseudo)spin or charge, upon external impulses or other environmental factors. The knowledge of the system’s relevant timescales and its dynamical behavior is also important for studying electronic transport through the nanostructures ^3, 4^. Moreover, the insight into dynamical processes is especially interesting from pure physical point of view, concerning the understanding of mechanisms of various phenomena, such as the formation of correlated states^5–11^, dynamical phase transitions ^12^, effects of decoherence and dissipation ^13^ or even the time crystals ^14^.

A principal example of a quantum impurity system in condensed matter and nanoscience is the Kondo system^15^, consisting of a localized magnetic moment coupled to metallic continuum of states. At sufficiently low temperatures, the spin-flip scattering of conduction electrons at the impurity’s site results in the formation of a correlated Kondo state, in which the magnetic moment is screened^16^. The electronic and transport properties of such system were initially explained using the effective low-energy bulk Kondo model ^15^, and then followed by the Wilson’s numerical renormalization group (NRG) method, allowing for a fully nonperturbative treatment ^17, 18^. The NRG method was later extended to the time domain ^19–22^ enabling the examination of evolution of electronic correlations and generally, nonequilibrium dynamics, which have recently become a very active field of interest.

The quantum impurity models are also relevant in the context of various artificial nanostructures, such as quantum dots or molecules, coupled to external leads, the behavior of which, in appropriate parameter space, reveals the Kondo physics. ^23–25^ Advanced nanofabrication techniques enable the implementation of devices in desired configuration or geometry, and with preferable number of subparts, such as dots or leads ^26–32^. This allows one to study the interesting local properties much more easily than in the case of bulk materials, where it is usually harder to decouple distinguished subsystem from large number of degrees of freedom or to design desired number of screening channels. Because the manipulation and tuning of local physical and chemical properties is more accessible in the case of such nanosystems ^27^, they could serve as a good basis to study general physical behavior, but also as a simulation of more complicated systems ^33^.

In this paper we in particular perform the numerical analysis of the quantum quench dynamics of the one-channel and two-channel Kondo models^34^. For this purpose, we employ the time-dependent numerical renormalization group (tNRG) approach^19–21^, implemented in the matrix product state formalism^22, 35^. More specifically, the one-channel Kondo model (1CK) effectively describes a single spin one-half $S=1/2$ impurity in bulk material or connected to external electronic reservoir, dynamical mean-field lattice models mapped on impurity model ^18^, adatoms on surfaces ^36, 37^, qubit in environment ^38, 39^, etc. On the other hand, multi-channel (two-channel in our case, 2CK) Kondo model describes single impurity connected to multiple independent bands ^34, 40, 41^ or could be effectively used to describe multiple impurity systems ^42–48^. Mapping of 1CK model in the presence of external periodic electric field also results in an effective 2CK model ^49^. Usually, one-channel Kondo model exhibits Fermi liquid behavior, with typical energy scale defined by the Kondo temperature $T_K$^16^. One the other hand, in the two-channel Kondo model there could be naturally more energy scales ^34, 45^, and exotic non-Fermi liquid behavior near critical points due to overscreening effects may emerge. Furthermore, if the channel symmetry in 2CK is broken, quantum phase transitions could occur ^50^.

**Figure 1:**
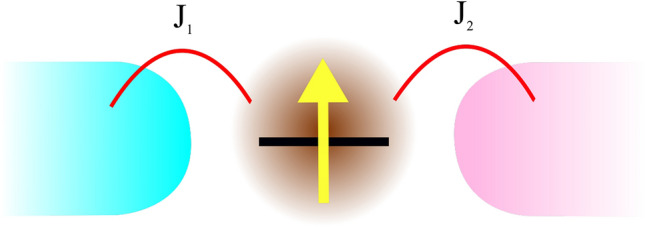
Schematic of a spin-1/2 magnetic impurity coupled to two screening channels via effective exchange interaction $J_\alpha$, where $\alpha =1$ ($\alpha =2$) for the first (second) channel.

Quantum quenches are possible way to change the Hamiltonian in time resulting in modification of original electronic structure and the occurrence of dynamics between the initial and final Hamiltonians ^21^. The goal of the present work is to shed more light onto the dynamical properties of $S=1/2$ impurity exchange-coupled to one or two conduction channels, as schematically illustrated in Fig. 1. In particular, we determine the time-evolution of the Loschmidt echo as well as the expectation value of the impurity’s spin, following quenches performed in the exchange coupling to external reservoirs, both in the absence and presence of magnetic field. Our numerical analysis indicates that the decay of the Loschmidt echo strongly depends on the type of quench and the ground state of the system, while the behavior of the impurity’s magnetization helps in identification of the relevant time scales in the system’s dynamics.

## Results

The general schematic of the studied system is shown in Fig. 1. It consists of a spin-1/2 impurity exchange-coupled to two screening reservoirs through the couplings $J_1$ and $J_2$, respectively. The Hamiltonian of the system is given by

1 $$\begin{aligned} H = \sum _{\alpha \mathbf{k} \sigma }\varepsilon _{\alpha \mathbf{k} \sigma } c^\dagger _{\alpha \mathbf{k} \sigma } c_{\alpha \mathbf{k} \sigma } + \frac{1}{2} \sum _{\alpha \sigma \sigma '}J_{\alpha } \mathbf {S} \psi _{\alpha \sigma }^{\dagger } \varvec{\sigma }_{\sigma \sigma '}\psi _{\alpha \sigma '} + B_zS_z, \end{aligned}$$

where $c^\dagger _{\alpha \mathbf{k} \sigma }$ ($c_{\alpha \mathbf{k} \sigma }$) denotes the creation (annihilation) operator of an electron with momentum $\mathbf{k}$, spin $\sigma$ and energy $\varepsilon _{\alpha \mathbf{k} \sigma }$ in channel $\alpha$. $J_\alpha$ denotes the exchange coupling between the impurity’s spin $\mathbf {S}$ and the conduction electrons in the channel $\alpha$ described by the respective field operators $\psi _{\alpha \sigma }^{\dagger }$, while $\varvec{\sigma }$ stands for the vector of Pauli spin matrices. Finally, the last term takes into account the Zeeman splitting, where $B_z$ is the external magnetic field applied locally to the impurity and expressed in units of $g\mu _B\equiv 1$, while $S_z$ describes the *z*-th component of the impurity’s spin.

We are interested in studying the dynamics of the system after a sudden quench (at time $t=0$) in the initial Hamiltonian $H_0$, as described by

2 $$\begin{aligned} H(t) = \theta (-t)H_0 + \theta (t)H, \end{aligned}$$

where $\theta (t)$ is the Heaviside step function. It is important to note that both initial ($H_0$) and final Hamiltonians (*H*) are in fact given by Eq. (1), the only difference is associated with a sudden change of model parameters, which happens at $t=0$. An important quantity describing the ability of the system to depart from its initial state after quenching is the Loschmidt echo, which is defined as ^12, 51–53^,

3 $$\begin{aligned} L(t) = |\langle \psi _0|\psi (t)\rangle |^2 = |\langle \psi _{0}|e^{-iHt}|\psi _{0}\rangle |^2. \end{aligned}$$

Here, $|\psi _{0}\rangle$ denotes the initial state, which is taken as the ground state of the Hamiltonian $H_0$, and then time-evolved according to the final Hamiltonian *H*. We note that in the case of a degenerate ground state (as in the case in the absence of magnetic field), an appropriate linear combination of states is taken as the initial state for time evolution. In fact, the form of evolution and decay of the Loschmidt echo gives an information about changing (in time) of fidelity of the final state with respect to the initial one. ^12^ For relatively weak quenches, when the system is being pushed to final state not so distant from its initial state, one expects that the echo function will be close to 1 in short time scales. In the opposite case, for large quenches, the echo function should more rapidly decay, meaning that system is getting far away from its initial state. In the following we examine the behavior of the Loschmidt echo in the case of (i) one-channel Kondo model ($J_1\equiv J$, $J_2=0$) and (ii) two-channel Kondo model (finite $J_1$ and $J_2$). Let us start with the former case.

### Quench dynamics in the case of one-channel Kondo model

**Figure 2:**
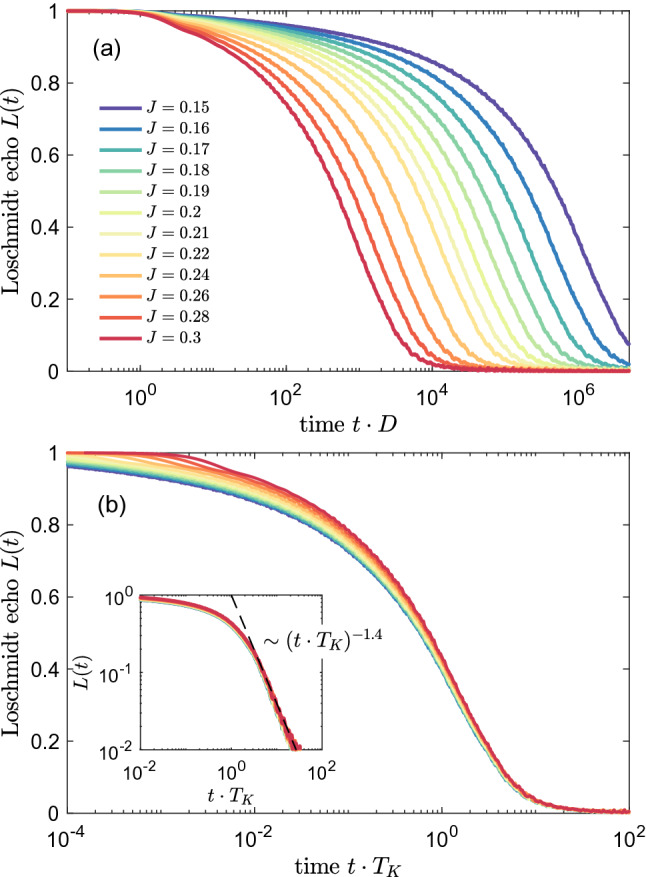
(**a**) The Loschmidt echo *L*(*t*) for quantum quench in the coupling strength $J_1\equiv J$, starting from $J=0$ to the value shown in the legend, while $J_2=0$. (**b**) The Loschmidt echo plotted vs time rescaled with the Kondo temperature $T_K$ determined from the halfwidth at half maximum of the impurity’s spectral function. The inset presents the universal behavior for $t\cdot T_K>1$, where $L(t)\sim (t\cdot T_K)^{-1.4}$. We have used the following NRG parameters: number of states kept at each iteration $N_K=2048$ and the band discretizaton parameter $\Lambda =1.3$, while *J* is expressed in terms of band halfwidth $D\equiv 1$.

In Fig. 2 we present the Loschmidt echo for quench in the coupling strength $J_1\equiv J$, when $J_2=0$, starting from $J=0$, which corresponds to a decoupled system, to different values of *J*, as listed in the legend. In this setup we are able to explore the dynamics of the system when the Kondo correlations set in. The figure was calculated by assuming the band discretization parameter $\Lambda =1.3$, which assures that the dependence of *L*(*t*) is sufficiently smooth. (For larger values of $\Lambda$ some artifacts due to discretization may be revealed, however, the general behavior is the same). Generally, in all the cases the Loschmidt echo is monotonically dropping to 0 in sufficiently long time scales, and for increasing value of final *J*, *L*(*t*) is decaying faster.

In the case of the one-channel Kondo model there is only one energy scale associated with the Kondo correlations—the Kondo temperature $T_K$, and the corresponding time scale is $t_K=1/T_K$. This time scale can be clearly seen in Fig. 2a, where lowering of *J* results in a decrease of $T_K$ and, thus, in an increase of $t_K$. In the following we will use the value of $J=0.2$ as a reference one in further analysis. For $J=0.2$, we find $T_K \approx 4\cdot 10^{-5}$, as estimated from the halfwidth at half maximum of the composite fermion operator spectral function. One can see that for our reference case the echo function starts to decay rapidly in short time scales, and as the time elapses the decay rate drops significantly. For time *t* before Kondo time $t_K\approx 2.5\cdot 10^4$, the dependence of *L*(*t*) is quite smooth and resembles a rapidly decaying polynomial function ^53^, whereas for times larger than $t_K$, the decay of *L*(*t*) slows down. (Note the logarithmic time scale in Fig. 2). A similar dependence is obtained for different values of final *J*. The only difference is the time needed to obtain comparable drop of the echo function, what is a consequence of the fact that for smaller quenches the system needs longer time to form fully correlated Kondo singlet state.

The similarity between different curves presented in Fig. 2a suggests a universality of *L*(*t*). This is explicitly demonstrated in Fig. 2b, which shows that the Loschmidt echo is a universal function of $t\cdot T_K$ in the long time limit $t\cdot T_K\gg 1$, corresponding to the low energy fixed point of the Kondo model, i.e., to the Kondo regime. Moreover, we numerically estimate the dependence of the Loschmidt echo on time and find that, $L(t)\sim (t\cdot T_K)^{-1.4}$, for $t\cdot T_K\gg 1$, see the inset of Fig. 2b. It is interesting to note that a similar algebraic dependence, but with a different exponent, have been predicted for interacting resonant level model^54^.

**Figure 3:**
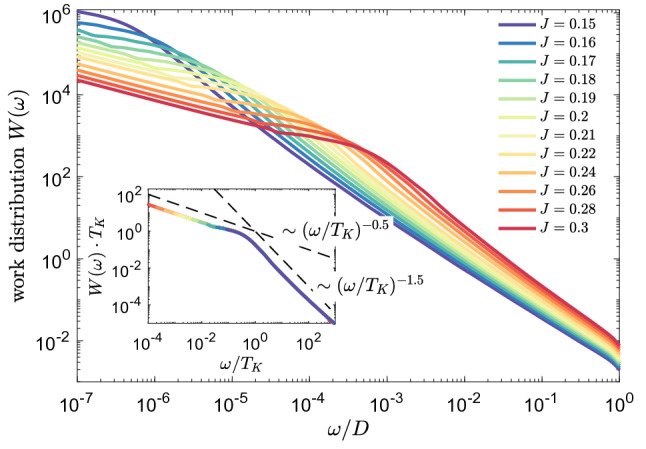
The work distribution calculated for quantum quench in the coupling strength $J_1\equiv J$, starting from $J=0$ to the value shown in the legend, while $J_2=0$. The inset presents the rescaled work distribution $W(\omega )\cdot T_K$ plotted as a function of $\omega / T_K$. We find the scaling $W(\omega )\sim \omega ^{-0.5}$ for $\omega <T_K$ and $W(\omega )\sim \omega ^{-1.5}$ for $\omega > T_K$. The other parameters are the same as in Fig. 2.

To provide more complete picture, we have also analyzed the energy dependence of the work distribution $W(\omega )$, which is defined as^55^

4 $$\begin{aligned} W(\omega ) = \sum _n \delta (\omega - E_n+E_0)|\langle \psi _n|\psi _{0}\rangle |^2, \end{aligned}$$

where $|\psi _n\rangle$ is the eigenstate of the final Hamiltonian with the corresponding eigenenergy $E_n$, while $E_0$ denotes the ground state energy of the initial Hamiltonian. The work distribution is closely related to the amplitude of the Loschmidt echo through the Fourier transform. We have computed $W(\omega )$ by collecting the data in logarithmic bins with respect to the zero energy. Such discrete data have been then broadened to obtain a continuous function, as in standard NRG calculations of correlation functions^56^. The work distribution, for the same type of quench as shown in Fig. 2, is displayed in Fig. 3. On the other hand, the inset presents $W(\omega )\cdot T_K$ plotted vs $\omega /T_K$, where a collapse of all the curves onto a single one can be observed. As can be seen, the work distribution exhibits a maximum at low energies and then decreases with energy as $W(\omega )\sim \omega ^{-0.5}$ till $\omega \approx T_K$. For larger energies, $\omega > T_K$, the slope of $W(\omega )$ is changed to $W(\omega )\sim \omega ^{-1.5}$. Thus, the dependence of rescaled work distribution $W(\omega )\cdot T_K$ clearly shows a universal behavior on $\omega /T_K$, similar to the Loschmidt echo.

**Figure 4:**
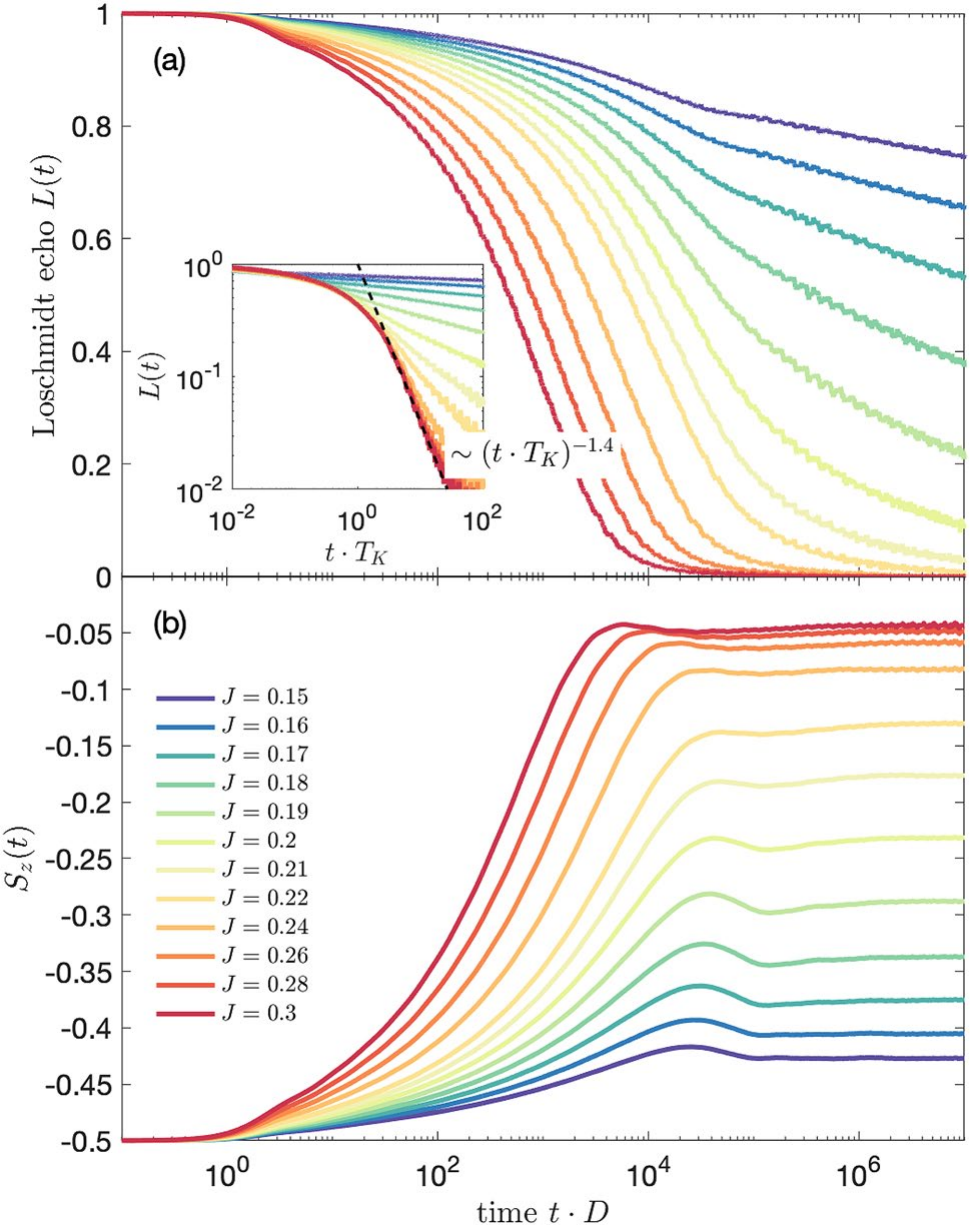
(**a**) The Loschmidt echo *L*(*t*) and (**b**) the evolution of the expectation value of the impurity’s spin $S_z(t)$ for the quench in $J_1\equiv J$ starting from 0 to value in given in the legend, with $J_2=0$ and assuming a finite magnetic field $B_z=4\cdot 10^{-5}$ applied to the system. The inset in (**a**) presents *L*(*t*) plotted as a function of $t\cdot T_K$. The other parameters are the same as in Fig. 2.

Let us now analyze the dynamics of the system in the presence of an external magnetic field. In the case of finite magnetic field a new energy scale occurs associated with external magnetic field $B_z$ ^29^, with the corresponding time $t_B=1/B_z$. The behavior of the system depends then on the ratio of $B_z$ and $T_K$. Note that $B_z$ corresponds directly to the Zeeman energy, cf. Eq. (1). In Fig. 4 we present the evolution of the Loschmidt echo and the expectation value of the impurity’s spin $S_z(t)$ for the quench performed in the coupling strength $J_1\equiv J$, with $J_2=0$, assuming that magnetic field $B_z=4\cdot 10^{-5}$ is applied to the system. The value of the field is equal to the Kondo temperature for $J=0.2$, thus the case of $J>0.2$ corresponds to $T_K>B_z$, while the case of $J<0.2$ displays the situation when the magnetic field is larger than the Kondo energy scale.

For large values of coupling $J>0.2$, one can see that the Loschmidt echo exhibits the dependence as if in the absence of magnetic field, cf. Figs. 2a and 4a. This is because $T_K\gg B_z$ and, consequently, $t_B\gg t_K$. The situation changes when $T_K$ is decreased and becomes of the order of $B_z$, which happens for values of final *J* very close to $J=0.2$. Then, the exchange coupling term (responsible for the Kondo correlations) competes with the Zeeman splitting term, which tends to suppress and split the Kondo peak. Because of that, the Loschmidt echo looses its universal time-dependence, see the inset in Fig. 4a, and its drop becomes much slower as the time increases. Eventually, for $J<0.2$, the decay of the Loschmidt echo is logarithmic in the long time limit.

The time scale $t_B$ associated with the magnetic field is also clearly visible in the time dependence of $S_z(t)$, which is shown in Fig. 4b. In the case of $B_z=0$, the local magnetic moment of the impurity is equal to zero for the whole time domain for any value of quench in *J*. Adding a nonzero magnetic field $B_z$ polarizes the impurity’s spin and produces a nonzero value of average magnetic moment at the initial time, i.e. when the system is decoupled ($J=0$), with $S_z(0)=-1/2$. Upon subsequent turning on of the coupling between the impurity and conduction electrons, the value of $S_{z}(t)$ increases due to the growing Kondo correlations in the system.

For $J<0.2$, the spin expectation value exhibits a small local maximum at time $t_B$, before establishing final nonzero value. On the other hand, for $J\approx 0.2$, $S_z(t)$ displays the largest local maximum, which is then followed by a small minimum, until the spin stabilizes its value at similar time scale as in the case of $J<0.2$. This behavior can be explained as follows. The two Hamiltonian terms (Kondo coupling and Zeeman term) are now of equal strength and are competing with each other. Thus, the impurity’s spin takes a value in-between two limiting cases: $S_z=0$ in the strong Kondo regime and $S_z=-1/2$ when only the magnetic field is present ($J=0$). Such competition and indeterminacy of the final state usually results in oscillations of the corresponding echo function. Consequently, the nonmonotonic dependence of $S_{z}(t)$ (local maximum followed by a local minimum before the flattening of function takes place) results from the existence of two competing time scales $t_K$ and $t_B$, corresponding to $T_K$ and $T_B$, which are of comparable order.

**Figure 5:**
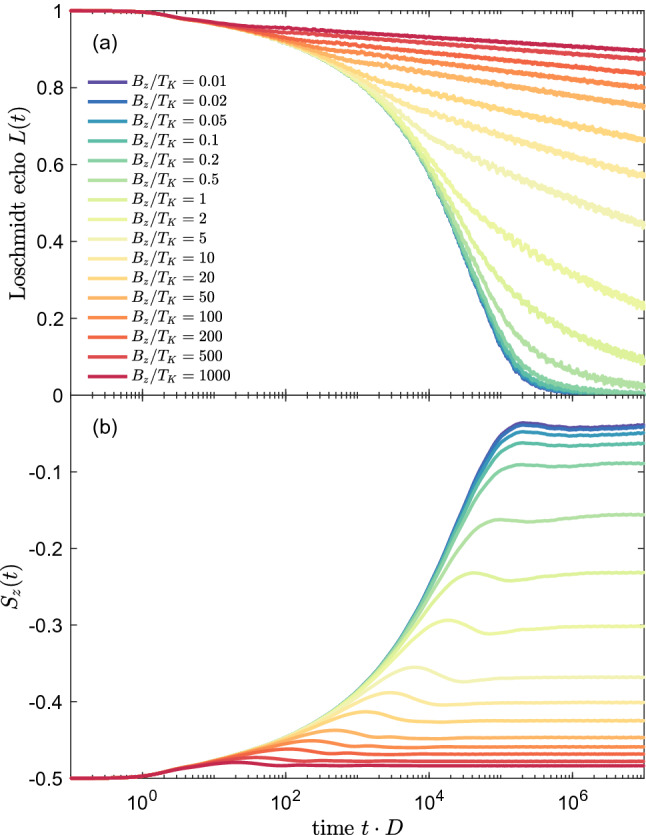
The time-evolution of (**a**) the Loschmidt echo and (**b**) spin expectation value calculated for quantum quench in $J_1\equiv J$, starting from $J=0$ to $J=0.2$, while $J_2=0$, for different values of magnetic field $B_z$, as listed in the legend. The magnetic field is expressed in units of the Kondo temperature for $J=0.2$, $T_K=4\cdot 10^{-5}$. The other parameters are the same as in Fig. 2.

The growth of $S_{z}(t)$ up to time $t_K$ is due to the development of the Kondo correlations in the system. For longer times $t\gtrsim t_K$, there is a local extremum (corresponding to change of slope of the Loschmidt echo), followed by flattening of functions when the final correlated state is obtained and local observables are fixed at some values. The local maxima are pinned at time corresponding to $t_B$, what reveals the effect of magnetic field. When the Kondo correlations are stronger than the Zeeman energy, i.e. for $J> 0.2$, the $S_{z}(t)$ function is increasing more rapidly reaching final value near 0 at time of the order of $t_K$ and the local maximum visible for $J\lesssim 0.2$ is vanishing since the scale $t_B$ is getting irrelevant. Only in these cases the vanishing local maxima are shifted to smaller time scales, emphasizing the crucial role of increased strength of Kondo coupling, leading to faster and more effective formation of the fully screened Kondo singlet state.

Figure 5a presents the Loschmidt echo for quench in the coupling strength from $J=0$ to $J=0.2$ for different values of applied magnetic field $B_z$ (each being kept constant during separate quenches). For magnetic fields smaller than the Kondo temperature corresponding to $J=0.2$, $B_z<T_K$, the echo functions drop to zero, meaning that magnetic field hardly affects the dynamical behavior of the system. This is because the time scale associated with magnetic field is much larger than $t_K$ and is thus almost irrelevant. When the two time scales are of the same order, which happens for $B_z\approx T_K$, a slow-down of the decay of Loschmidt echo is observed and *L*(*t*) takes nonzero values in the considered time scale. Furthermore, for larger values of $B_z$, the echo functions are decaying much slower due to the dominant role of magnetic field and the fact that $t_B\ll t_K$. In fact, for very large $B_z$, the Loschmidt echo becomes approximately flat and very weakly decays with time. This implies that the quench in *J* does not overcome the effect of large magnetic field and the impact of quenching is effectively much reduced. We note that by the final value theorem one can relate the long time behavior of the Loschmidt echo $L(t\rightarrow \infty )$ to $W(\omega \rightarrow 0)$, which is negligible in the considered cases. Consequently, it implies that although the decay of *L*(*t*) is much slowed down, the echo function approaches zero in the long time limit, $L(t\rightarrow \infty )\rightarrow 0$.

The corresponding time-evolution of the spin expectation value is displayed in Fig. 5b. In the behavior of $S_z(t)$ one can nicely see the growth of $t_B$ as the magnetic field is reduced, which is revealed in the dependence of the local maximum visible in $S_z(t)$ on $B_z$. When, however, $t_B>t_K$, the position of the maximum saturates and stays approximately at $t_K$. Moreover, decreasing the value of magnetic field results in lowering of the impurity’s magnetization. Once $B_z<T_K$, i.e. $t_B>t_K$, $S_z(t)$ becomes almost fully suppressed due to the development of the Kondo singlet state in the long time limit.

**Figure 6:**
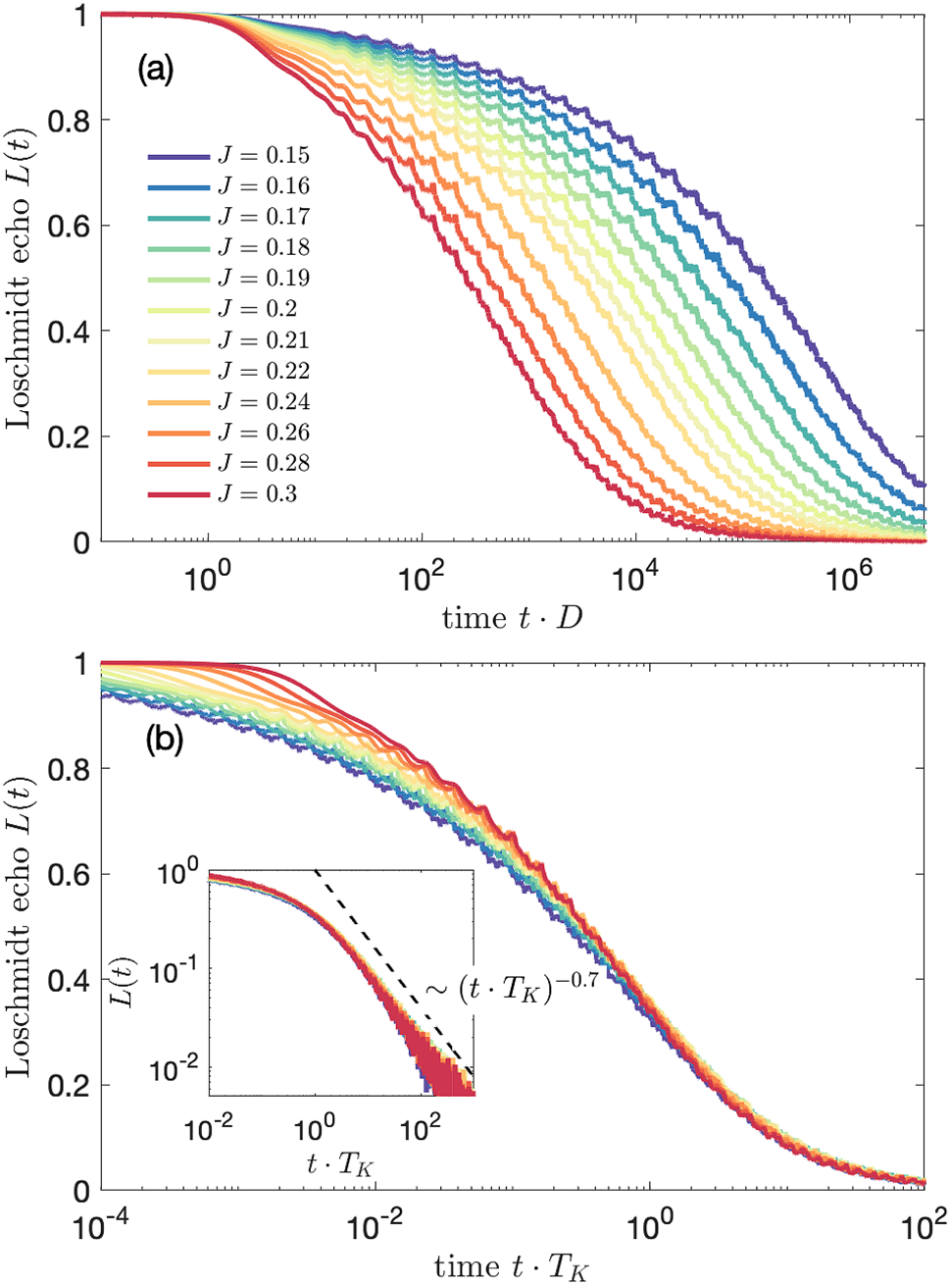
(**a**) The Loschmidt echo *L*(*t*) for quantum quench in the coupling strength $J_1=J_2\equiv J$, starting from $J=0$ to the value shown in the legend. (**b**) The Loschmidt echo plotted vs time rescaled with the Kondo temperature $T_K$, with $T_K=D\exp (-1/J\rho )$. The inset presents the universal behavior for $t\cdot T_K>1$, where $L(t)\sim (t\cdot T_K)^{-0.7}$. In calculations we have used the following NRG parameters: $N_K=14000$ and $\Lambda =1.6$.

### Quench dynamics in the case of two-channel Kondo model

**Figure 7:**
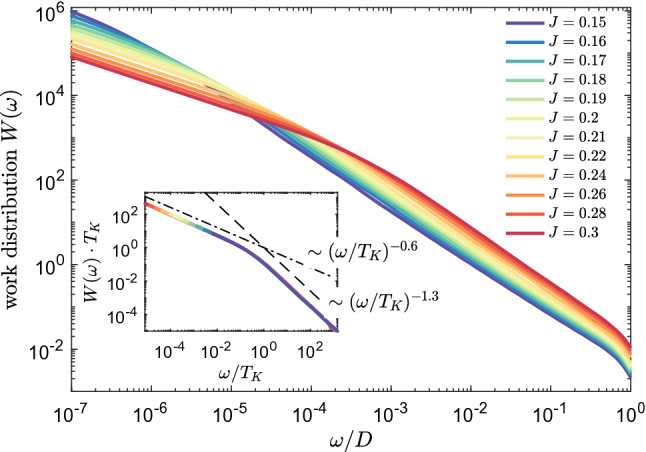
The work distribution calculated for quantum quench in $J_1=J_2\equiv J$, starting from $J=0$ to the value shown in the legend. The inset presents the rescaled work distribution $W(\omega )\cdot T_K$ plotted as a function of $\omega / T_K$. We find the scaling $W(\omega )\sim \omega ^{-0.6}$, for $\omega <T_K$, and $W(\omega )\sim \omega ^{-1.3}$, for $\omega > T_K$. The other parameters are the same as in Fig. 6.

**Figure 8:**
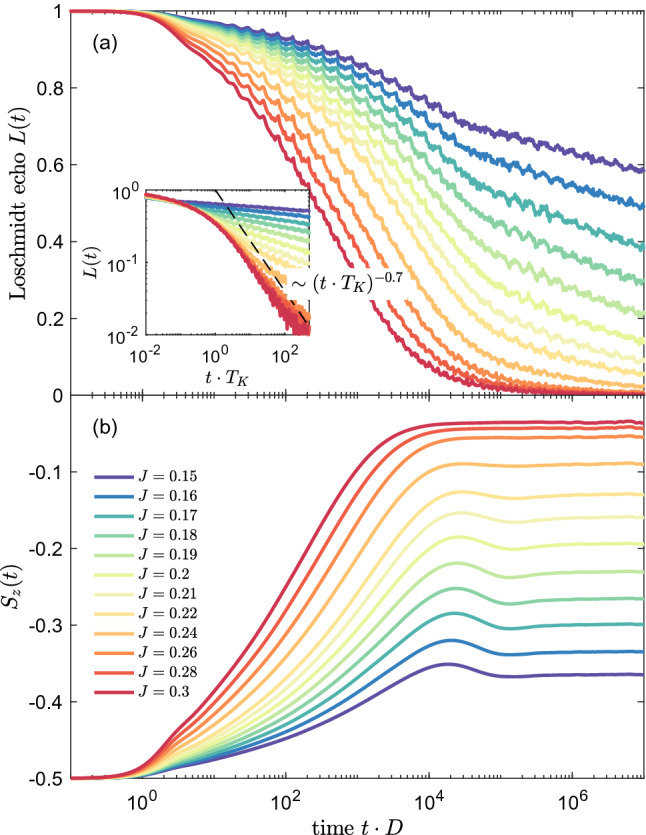
(**a**) The Loschmidt echo *L*(*t*) and (**b**) impurity’s spin expectation value for quantum quench in the coupling strength $J_1=J_2\equiv J$, starting from $J=0$ to the value shown in the legend in the presence of external magnetic field $B_z=4\cdot 10^{-5}$. The inset in (**a**) presents *L*(*t*) plotted as a function of $t\cdot T_K$. The other parameters are the same as in Fig. 6.

Let us now consider the case of magnetic impurity coupled to two conducting channels, i.e. when both $J_1$ and $J_2$ are finite. Figure 6 presents the time evolution of the Loschmidt echo for quench performed in $J_1=J_2\equiv J$ starting from $J=0$. In other words, the system evolves from the initial state of decoupled impurity to the final state of impurity equally coupled to two conduction bands. Such final state is an example of a non-Fermi liquid state, in which two channels try to screen the spin giving rise to an exotic ground state of the system. The calculations in the case of the two-channel Kondo model have been performed for $N_K=14000$ and $\Lambda =1.6$. We note that some small oscillations visible in *L*(*t*) are exclusively due to artifacts associated with discretization of conduction band. Similarly to the single-channel case studied in previous section, the decay of the Loschmidt echo depends on the magnitude of *J*, and *L*(*t*) becomes suppressed at earlier times as *J* grows. This is quite intuitive, since the system needs more time to develop the Kondo state when the exchange couplings are small. Nevertheless, once such state is formed, *L*(*t*) drops to zero indicating that the final state is orthogonal to the initial one. Interestingly, when one rescales the Loschmidt echo with $T_K=D\exp (-1/J\rho )$, it can be seen that for $t>1/T_K$ the curves collapse onto a single curve, demonstrating a universal behavior. Moreover, contrary to the single-channel case displayed in Fig. 2, we now find that the decay of the Loschmidt echo is slowed down and can be described by, $L(t)\sim (t\cdot T_K)^{-0.7}$, see the inset of Fig. 6b. One can also search for a universality in the behavior of the work distribution, which is presented in Fig. 7. When rescaled with the Kondo temperature, $W(\omega )$ indeed exhibits a universal behavior. In particular, from our numerical analysis we find the scaling, $W(\omega )\sim \omega ^{-0.6}$, for $\omega <T_K$ and $W(\omega )\sim \omega ^{-1.3}$, for $\omega > T_K$, see the inset of Fig. 7.

Let us now consider the dynamical behavior of the system in the presence of external magnetic field. We again consider the quench from $J_1 = J_2=0$ to the non-Fermi liquid fixed point, i.e. $J_1 = J_2$, however, assuming that the impurity is subject to $B_z=4\cdot 10^{-5}$, which is of the order of the Kondo temperature for $J=0.2$. The Loschmidt echo for this situation is presented in Fig. 8a, where the inset displays the dependence of *L*(*t*) as a function of rescaled time. Consider first the case of quench when the Kondo time scale $t_K$ is much smaller than the time scale associated with finite magnetic field $t_B$, i.e. $J<0.2$. One can see that *L*(*t*) decays very slowly and it changes slope around $t\approx t_B$. This is associated with the fact that magnetic field dominates over the Kondo correlations and the initial and final states are not far from each other. On the other hand, the decay of the Loschmidt echo is much more pronounced when $J>0.2$, i.e. when the Kondo correlations are larger than the induced Zeeman splitting. Then, we observe that the time dependence of *L*(*t*) resembles that in the absence of magnetic field. Indeed, for $J=0.3$, we recover the long-time behavior with $L(t)\sim (t\cdot T_K)^{-0.7}$, see the inset of Fig. 8a.

The corresponding dynamics is also revealed in the time dependence of the spin expectation value, which is shown in Fig. 8b. In the initial state the impurity is fully spin-polarized, $S_z(0)=-1/2$, and the polarization starts to decrease in the course of time evolution due to the development of Kondo correlations. This decrease continues until $t\approx t_B$, when the magnetization saturates and attains a time-independent value. For example, in the case of $J=0.15$, the spin expectation value becomes reduced to $S_z(t>t_B)\approx -0.35$. Nonetheless, for large enough *J*, the impurity’s spin may become almost fully compensated by the Kondo correlations, see e.g. the case of $J=0.3$ in Fig. 8b.

**Figure 9:**
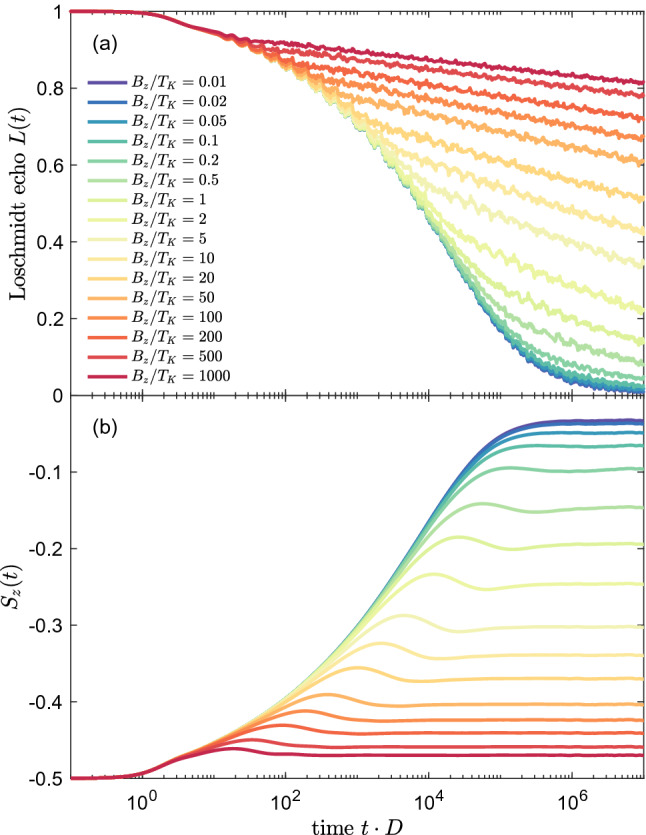
(**a**) The Loschmidt echo *L*(*t*) and (**b**) impurity’s spin expectation value for quantum quench in the coupling strength $J_1=J_2\equiv J$ from $J=0$ to $J=0.2$ in the presence of external magnetic field indicated in the legend. Magnetic field is expressed in units of $T_K=4\cdot 10^{-5}$. The other parameters are the same as in Fig. 6.

In Fig. 8 we have inspected the case when the magnetic field is fixed, however, it is also interesting to examine the situation when the quench is performed to the same Kondo state, from $J=0$ to $J=0.2$, but magnetic field takes different values, yet still constant during the whole time evolution. This is presented in Fig. 9, which shows the time dependence of both *L*(*t*) and $S_z(t)$ for different strengths of magnetic field, as indicated. Clearly, the decay of wave function overlap becomes slower and slower as the magnetic field grows. When $B_z/T_K \ll 1$, the Loschmidt echo drops quickly in accordance with $L(t)\sim (t\cdot T_K)^{-0.7}$. However, for $B_z/T_K \gg 1$, the echo function exhibits only a very slow suppression as the time goes by, which reflects the fact that the Kondo correlations are suppressed by strong magnetic field, see Fig. 9a. These observations are corroborated by the time dependence of the impurity’s spin, which is presented in Fig. 9b. The magnetic impurity becomes highly polarized for $B_z \gg T_K$ and the impurity’s magnetization drops as the magnetic field becomes lowered. As a consequence, for $B_z\ll T_K$, the initial magnetization becomes almost fully counterbalanced by the Kondo correlations in the long time limit.

**Figure 10:**
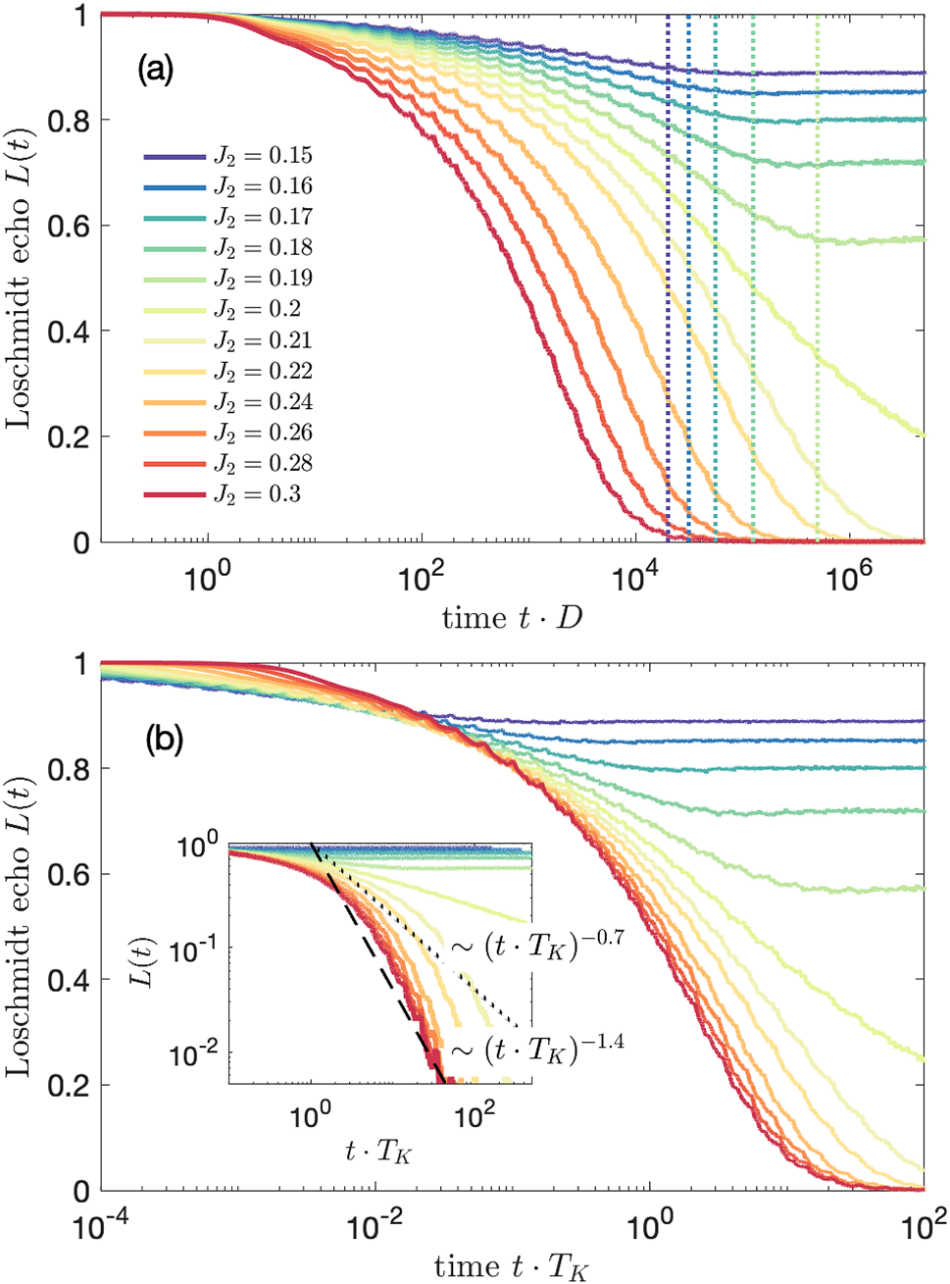
(**a**) The Loschmidt echo *L*(*t*) for quantum quench in the coupling strength $J_2$ from $J_2=0$ to the value shown in the legend, with fixed $J_1=0.2$. (**b**) The Loschmidt echo plotted vs time rescaled with the Kondo temperature $T_K$, where $T_K \approx D \exp (-1/\rho J_2)$. The vertical dashed lines in (**a**) indicate the time scale $t^*= \alpha /T^*$ for $J_2<0.2$, where $\alpha$ is a numerical factor of the order of unity. The other parameters are the same as in Fig. 6.

Up to now, we have studied the case when the quench was performed in both exchange couplings at the same time, i.e. the evolution was from the decoupled system to the case with $J=J_1=J_2$, when the final state was the non-Fermi liquid state of the two-channel Kondo problem. It is however also interesting to examine the situation when the initial state of the system is the usual Kondo state, as given by the single-channel Kondo model, while the finite state may be different. We thus assume $J_1=0.2$ for both the initial and final Hamiltonians, whereas the quench is performed in $J_2$, starting from $J_2=0$ to a finite value. The corresponding Loschmidt echo for such scenario is presented in Fig. 10. It is also important to note that in the case of finite channel anisotropy, $J_1\ne J_2$, there exist an additional energy scale $T^*\sim (J_1-J_2)^2$, which borders the Fermi liquid phase from crossover regime to the non-Fermi liquid phase ($J_1=J_2$), at which $T^*$ vanishes ^45^. Consequently, one may expect that a new time scale $t^*$, associated with $T^*$, would be relevant for the system dynamics. Indeed, as we show in the sequel, the time evolution of the Loschmidt echo exhibits some signatures of the channel anisotropy time scale $t^*\sim 1/T^*$.

When the quench is performed for $J_2<J_1$, the echo function exhibits a slow decay as the time increases. Interestingly, one can observe a change of slope of *L*(*t*) at the time scale approximately corresponding to $t^*\sim 1/T^*$. After this time, the Loschmidt echo attains a value, which rather weakly depends on time, see Fig. 10a. Note that the characteristic time scale, $t^*= \alpha /T^*$, where $\alpha$ is a numerical factor of the order of unity, is marked in Fig. 10a with vertical dotted lines. This basically indicates that the time-evolved state is dominated by the Fermi liquid phase of impurity coupled to the first conduction channel. A completely different situation occurs for $J_2>J_1$. Then, the initial and final states are orthogonal in the long time limit. This is because the initial state is formed by the impurity coupled to the first channel, while the final state comprises the impurity predominantly coupled to the second channel. Thus, in the course of evolution, for large $J_2$, the Fermi liquid state is transferred from a one-channel Kondo state with the first screening channel to a one-channel Kondo state with the second channel. Moreover, during the evolution, for $t_K<t<t^*$, the system needs to pass through the non-Fermi liquid phase. This can be recognized both in Fig. 10b and in the corresponding inset for intermediate times. We also note that for long times the time dependence of *L*(*t*) becomes similar to that in the single channel case, see the inset of Fig. 10b. On the other hand, the case of $J_1=J_2$ corresponds exactly to the quench from the Fermi liquid to the non-Fermi liquid state. For this situation, one can see that the Loschmidt echo decays much slower as compared to the case of $J_2>J_1$, and there is no upturn as in the case of $J_2<J_1$. Instead, one observes a steady decrease, similarly as in the quench presented in Fig. 6, though the time dependence is not exactly the same, cf. the inset of Fig. 10b.

**Figure 11:**
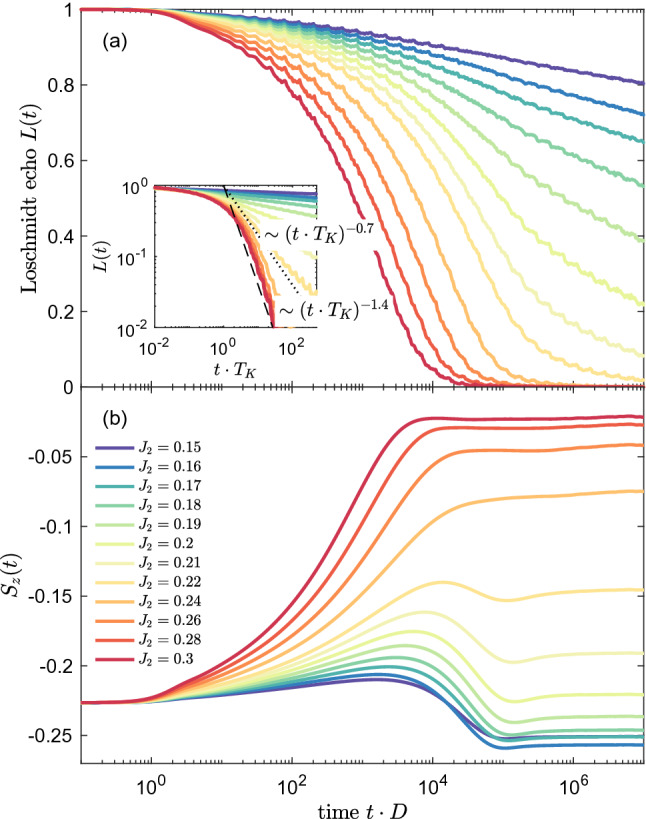
(**a**) The Loschmidt echo *L*(*t*) and (**b**) $S_z(t)$ for quantum quench in the coupling strength $J_2$ from $J_2=0$ to the value shown in the legend, with fixed $J_1=0.2$ and in the presence of magnetic field $B_z=4\cdot 10^{-5}$. The inset presents *L*(*t*) plotted vs time rescaled with the Kondo temperature $T_K$. The other parameters are the same as in Fig. 6.

The same type of quench as presented in Fig. 10 but performed in the presence of a constant magnetic field is shown in Fig. 11. At initial time, the impurity is in a spin-split Kondo state, where $B_z\approx T_K$, such that the corresponding impurity spin expectation is half-suppressed $S_z(0)\approx -0.23$, see Fig. 11b. For relatively weak quenches, $J_2<0.2$ one can see that the decay of the Loschmidt echo is rather slow, see Fig. 11a. Moreover, for $t>t_B$, the impurity’s magnetization becomes increased to $S_z(t>t_B)\approx -0.25$, which signals the fact that Kondo correlations become slightly weakened by the second weakly-coupled channel. Note that a similar behavior has been also found in the case of single-channel Kondo model, cf. Fig. 5. With increasing the magnitude of the quench, the decay of the Loschmidt echo becomes enhanced. Eventually, for $J_2>0.2$, *L*(*t*) drops quickly to zero indicating a completely different final state. This is a similar situation as in the absence of magnetic field for the quench with large enough $J_2$, cf. Fig. 10. As far as the impurity’s magnetization is concerned, with increasing $J_2$, the magnetization in the long time limit drops, which is an indication of Kondo correlations and the respective spin singlet state formation between the conduction band of the second channel and the impurity’s spin. The evolution of the Loschmidt echo decay with increasing the magnitude of quench is shown in the inset of Fig. 11. One can see that indeed the suppression of *L*(*t*) happens faster for larger $J_2$ and for $J_2=0.3$ it approaches $L(t)\sim (t\cdot T_K)^{-1.4}$, as in the single-channel case.

## Discussion

We have numerically studied the time dynamics of the one-channel and two-channel spin one-half Kondo models upon application of a quench in the underlying Hamiltonian. The calculations have been performed by means of the time-dependent numerical renormalization group method, within the matrix product state framework. This method allows for a non-perturbative inclusion of correlation effects and enables a very accurate description of the system’s dynamics. The focus of the paper has been put on the behavior of the Loschmidt echo, which describes the system’s ability to change its state during the time evolution, after the quench in the strength of coupling to the two conduction channels. We have also analyzed the effect of external magnetic field on the quench dynamics, calculating in addition the time dependence of the impurity’s magnetization.

In the case of the one-channel Kondo model, for quenches in the absence of magnetic field, we have shown that the Loschmidt echo exhibits a universal behavior with the relevant time scale given by the inverse of the Kondo temperature, $t_K = 1/T_K$. Moreover, we have numerically estimated the decay rate and found that, $L(t)\sim (t\cdot T_K)^{-1.4}$, for $t>T_K$. This universality is also revealed in the work distribution, which scales as, $W(\omega )\sim \omega ^{-0.5}$, for $\omega <T_K$, and $W(\omega )\sim \omega ^{-1.5}$, for $\omega >T_K$. Next, we have examined the system’s dynamics in the presence of external magnetic field $B_z$. The initial state for the time evolution was the state of a fully spin polarized magnetic impurity, decoupled from screening channels. We have analyzed how the decay of the Loschmidt echo depends on the ratio of magnetic field and the relevant Kondo temperature. For stronger quenches, the echo function was found to follow the behavior predicted in the absence of magnetic field, while for weaker quenches, the decay of *L*(*t*) was slowed down. This behavior was also reflected in the time dependence of the impurity’s spin expectation value, which increases as the time goes by with the corresponding local extremum for times of the order of $t_B=1/B_z$.

Finally, we have considered the quench dynamics in the case of the two-channel Kondo model. First, we have studied the quenches from decoupled system to the final state with equal couplings to both conduction channels, i.e. to the non-Fermi liquid state. For such genuine situation, we have shown that the time dependence of *L*(*t*) for different values of final exchange couplings collapses onto a single universal curve for $t>t_K$, with $L(t)\sim (t\cdot T_K)^{-0.7}$. Furthermore, for the corresponding work function we have numerically estimated the scaling to be, $W(\omega )\sim \omega ^{-0.6}$, for $\omega <T_K$, and $W(\omega )\sim \omega ^{-1.3}$, for $\omega >T_K$. We have also analyzed the time evolution of the Loschmidt echo and spin expectation value in the presence of constant magnetic field. Similarly to the single-channel case, we have found a slow-down of the Loschmidt echo decay at the time scale associated with magnetic field. Furthermore, we have determined the system’s dynamical behavior in the case of quench from an initial Fermi liquid state (as given by the impurity coupled to the first conduction channel) to a new final state obtained by turning on the coupling to the second screening channel. For such case, we have shown that the time evolution reveals a new time scale associated with the channel anisotropy.

## Method

We have studied the dynamical behavior of the considered Kondo models using the time-dependent numerical renormalization group method^19, 20^. The dynamics following sudden discrete quench (at time $t=0$) is described by the time evolution of the following general Hamiltonian

5 $$\begin{aligned} H(t) = \theta (-t)H_0 + \theta (t)H, \end{aligned}$$

where $\theta (t)$ is the step function, $H_0$ is the initial Hamiltonian of the system and *H* denotes the final system’s Hamiltonian.

Both Hamiltonians are solved with the Wilson’s numerical renormalization group method^17, 18, 35^. In this approach the conduction band is logarithmically discretized with parameter $\Lambda$ and mapped onto a tight-binding semi-infinite chain, while the impurity is coupled only to the first site ($n=0$) of the chain^17^. After this transformation, the Hamiltonian (1) becomes^17, 18^

6 $$\begin{aligned} H = \frac{1}{2} \sum _{\alpha }\sum _{\sigma \sigma '}J_{\alpha } \mathbf {S} f_{\alpha ,0\sigma }^{\dagger } \varvec{\sigma }_{\sigma \sigma '}f_{\alpha ,0\sigma '} +\sum _\alpha \sum _{n=0}^{\infty }\sum _{\sigma } t_{{\alpha }n}(f_{\alpha ,n\sigma }^{\dagger }f_{\alpha ,n+1\sigma } +f_{\alpha ,n+1\sigma }^\dag f_{\alpha ,n\sigma }) + B_zS_z, \end{aligned}$$

where $f_{\alpha ,n\sigma }^{\dagger }$ is the creation operator of a spin-$\sigma$ electron on Wilson site *n* in channel $\alpha$. The first term of the Hamiltonian describes the coupling of the impurity to electrons from conduction channel $\alpha$. The second term accounts for the metallic bands modeled as Wilson chains, where $t_{\alpha n}$ denotes the hopping between sites *n* and $n+1$ in the channel $\alpha$. Finally, the last term describes the local Zeeman energy.

Both initial and final Hamiltonians are solved in an iterative manner with $N_K$ lowest-energy eigenstates kept at each of *N* iterations. The discarded states and all states obtained on the last iteration of the procedure (labeled with superscript *D*) are used to create the complete initial and final eigenbases ^19^

7 $$\begin{aligned} \sum _{nse}|nse\rangle ^{\!D}_{0} \,^{D}_{\,0}\!\langle nse| \!=\! {\hat{\mathbbm{1}}} \;\;\;\, {\mathrm{and}} \,\;\;\; \sum _{nse}|nse\rangle ^{\!D} \,^D \!\langle nse| \!=\! {\hat{\mathbbm{1}}}. \end{aligned}$$

Here, all eigenstates are labeled with the following indices: *n* refers to the iteration number, *s* to a specific eigenstate and *e* describes the environmental part of the chain. To facilitate calculations, we have exploited the spin and charge symmetries of the system.

The dynamical quantities are determined in frequency domain. In order to obtain the relevant time dependencies, all collected Dirac delta peaks are Fourier transformed into the time domain

8 $$\begin{aligned} O(t)=\int _{-\infty }^{\infty }O(\omega )e^{-i \omega t} d \omega , \end{aligned}$$

where $O(t)=L(t), S_z(t)$. For further technical details see^20, 22^.

## Data availability

The datasets generated and analyzed during the current study are available from the corresponding author on reasonable request.
